# Essential health services delivery in Zimbabwe during the COVID-19 pandemic: perspectives and recommendations

**DOI:** 10.11604/pamj.supp.2020.35.143.25367

**Published:** 2020-08-11

**Authors:** Grant Murewanhema, Richard Makurumidze

**Affiliations:** 1 Department of Obstetrics and Gynaecology, University of Zimbabwe, College of Health Sciences, Zimbabwe,; 2 Department of Community Medicine, University of Zimbabwe, College of Health Sciences, Zimbabwe,; 3 Institute of Tropical Medicine, Antwerp, Belgium,; 4 Faculty of Medicine and Pharmacy, Free University of Brussels (VUB), Brussels, Belgium

**Keywords:** COVID-19, Zimbabwe, health services delivery, delays

## Abstract

Zimbabwe reported its first case of COVID-19 on 20 March 2020, and since then the number has increased to over 4000. To contain the spread of the causative SARS-CoV-2 and prepare the healthcare system, public health interventions, including lockdowns, were imposed on 30 March 2020. These resulted in disruptions in healthcare provision, and movement of people and supply chains. There have been resultant delays in seeking and accessing healthcare by the patients. Additionally, disruption of essential health services in the areas of maternal and child health, sexual and reproductive health services, care for chronic conditions and access to oncological and other specialist services has occurred. Thus, there may be avoidable excess morbidity and mortality from non-COVID-19 causes that is not justifiable by the current local COVID-19 burden. Measures to restore normalcy to essential health services provision as guided by the World Health Organisation and other bodies needs to be considered and implemented urgently, to avoid preventable loss of life and excess morbidity. Adequate infection prevention and control measures must be put in place to ensure continuity of essential services whilst protecting healthcare workers and patients from contracting COVID-19.

## Perspective

Zimbabwe confirmed its first COVID-19 case in March 2020 [1]. Since then the number has been increasing, and is now approximating 4000 [1]. A national lockdown was introduced on 30 March 2020. Apart from limiting human movements, the restrictions resulted in stoppage of non-emergency medical care at health institutions, and disruptions in supply chains of consumables for medical care. These disruptions could have devastating consequences on healthcare delivery for the population. Due to weak surveillance systems in Zimbabwe, the accurate indirect effects of the COVID-19 maybe difficult to assess. However, experience from previous crises such as the Ebola viral disease outbreak show that the indirect impacts of such disasters can be far reaching [2]. Preliminary data are revealing preventable losses of life that can be attributed indirectly to the COVID-19 pandemic. A comparative audit of maternal and perinatal outcomes at two hospitals in Harare suggested reduced utilisation of health services, and a trend towards a rise in maternal and perinatal mortality [3]. We review the implications of lockdowns on healthcare delivery and offer mitigating action-oriented public health interventions to limit further damage.

**The delays:** the three-delays model is a useful conceptual framework for examining causes of adverse outcomes in healthcare delivery [4]. It includes delays in referring patients appropriately and timeously, reaching health care facilities once appropriately referred, and obtaining appropriate treatment once at a referral facility. Though originally used to describe delays in maternity, it can be applied to general healthcare provision [5]. In the COVID-19 pandemic, first stage delays are occurring due to reduced consultations, attributable to lack of personal protective equipment (PPE) and fear of contracting COVID-19 by healthcare workers (HCWs). Second stage delays are occurring owing to challenges in transporting patients from primary level to higher-level facilities. Public transporters, except for those affiliated to a national passenger company are banned. Several checkpoints are in place to control movements. Bad roads, shortage of fuel and poor digital networks compound the delays. Maternity conditions are time-sensitive; thus, delays lead to excessive mortality. At referral facilities patients risk facing third delays. Supply chain disruptions have led to shortage of consumables, including life-saving medicines. In some places, attention has been shifted to COVID-19 related care and emergency-preparedness capacity building. The impact of the delays on mortality and morbidity will have a skewed distribution. People with comprehensive medical insurance schemes can access treatment services timeously in the private sector. Unfortunately, close to 90% of Zimbabweans have no medical insurance and will struggle to access even basic health services. Thus, people will die from preventable causes, especially in pregnancy. Zimbabwe´s maternal mortality is among the highest in Sub-Saharan Africa and could be worse by the end of the pandemic [6].

**Availability of supplies and PPE in health facilities:** many health facilities lack basic consumables including medicines and sundries. Some of these are imported, or the local industries stopped production temporarily due to lockdown. Shortages of intravenous fluids, gloves and essential medicines have been widely reported. Lack of access to PPE is another barrier to service delivery. Authorities have failed to supply appropriate PPE. Thus, HCWs have found it difficult to handle patients whose COVID-19 status is unknown. There have been multiple reports of patients being denied access to treatment without valid test results.

**Oncology and specialist services:** outpatient departments and elective surgical operations were stopped at the onset of the outbreak including for patients with cancers. Cancers can progress from early stage, operable disease to advanced, inoperable disease in a short time, which increases costs to patients and institutions, and reduces overall survival. Patients initially requiring less sophisticated treatments may eventually require more expensive, complex and toxic multimodality treatments. Disease progression is not limited to just cancers. By the time normal services resume, patients will have advanced diseases. Institutions were severely incapacitated at the beginning of the pandemic, and will be worse off when normal services resume, unless drastic measures are implemented to capacitate them.

**Maternal, sexual and reproductive health services:** obstetric problems requiring antenatal surveillance are neglected, and women with high-risk pregnancies are presenting in labour or with advanced complications. Delays are occurring in getting uterine evacuations after miscarriages, which can potentially result in overwhelming sepsis or fatal haemorrhage. The requirements for blood products have gone up as patients present to hospital with severe anaemia, yet blood stores are depleted. High school students are the usual donors but schools are closed and movements restricted. The National Blood Transfusion Services of Zimbabwe recently cited shortage of blood processing reagents due to disrupted supply chains and foreign currency shortages [7]. Disruption in supply chains, shortages of PPE and transport problems may interrupt or delay access to vaccinations in times of crisis, including the COVID-19 pandemic. Resurgence of measles cases occurred in the DRC during the recent Ebola outbreak [8]. At the beginning of the lockdown in Zimbabwe, many vaccination clinics were stopped, and some children possibly missed some doses, though services seem to be gradually normalising. Experience from previous outbreaks suggests that maternal, sexual and reproductive health services are easily neglected, resulting in reduced utilisation and undesirable maternal consequences [9,10]. Reported shortages of hormonal contraceptives and condoms in health facilities and quarantine centres may result in upsurge of unintended pregnancies and sexually transmitted infections, including HIV. Unintended pregnancies can lead to unsafe terminations, fatal sepsis, haemorrhage and mortality. Couples may currently be spending more time together due to lockdowns, with a resultant increase in the need for contraceptive products.

**Chronic diseases:** the population suffers from a high burden of chronic communicable and non-communicable diseases. Hypertension and diabetes are among the leading causes of morbidity for chronic non-communicable diseases. Among chronic communicable diseases, HIV leads with about 1.3 million individuals currently living with HIV/AIDS (PLWHA), and on antiretroviral therapy (ART) [11]. Chronic care for these conditions is affected by reduced outpatient attendances and shortages of medicines. Schedules for prescription refills and routine laboratory monitoring tests have been disrupted. Unfortunately, poorly controlled chronic diseases increase the odds of death from COVID-19.

**Recommendations:** the World Health Organisation (WHO) published guidance to support countries on how to maintain essential services during pandemics [12]. Several key recommendations are encompassed in the guidelines. Critical is contextualisation before adoption. Some of the strategies include prioritising essential health services and adapting to the changing context and needs, optimising service delivery settings and platforms, and establishing safe and effective patient flow at all levels. Additionally, they recommend rapidly optimising health workforce capacity, maintaining the availability of essential medicines, equipment and supplies, removing financial barriers to access of health services and strengthening communication strategies to support the appropriate use of essential health services. We recommend prioritisation of the development of local guidelines to prevent avoidable morbidity and mortality. Specifically, we offer the following action-oriented recommendations that we think are practical and implementable urgently (Table 1).

**Table 1:** recommendations for maintaining essential health services

Item	Recommendations	Priority
Phase 1 delays	Provide all HCWs at all levels of care with adequate PPE.	High
	Educate all HCWs at all levels of care on appropriate PPE for specified tasks.	
	Provide ongoing education to allay anxiety among HCWs, and work on dealing with myths and misconceptions.	
Phase 2 delays	Ensure availability of functional and fuelled ambulances at all levels of care.	High
	Restore urgently a functional and efficient public transport system with adequate IPC measures.	
	Remove the need for pregnant women to produce proof of their need to travel.	
Phase 3 delays	Restore urgently the supply chains for all essential medicines, sundries and equipment for all levels of healthcare.	High
	Prioritise allocation of foreign currency to the health sector and to ancillary services such as blood transfusion services.	
Availability of ppe and supplies	Adopt WHO guidance on rationalising the use of PPE in pandemic times.	High
	Encourage and support local production of PPE through local universities, technical colleges and industries.	
	Ensure efficient and appropriate, rational use of PPE at all levels of care.	
Oncology and specialist services	Urgently restore all outpatient services for patients requiring specialist care, with appropriate IPC measures, including physical distancing and reduced volumes of consultations per unit.	High
	Make telemedicine accessible to reduce the need for face-to-face consultations whenever possible.	
	Urgently restore elective surgeries, prioritising oncology patients, with minimal number of personnel in theatre, and optimal IPC measures.	
	Restore chemoradiotherapy services urgently, with strict IPC measures. Allocate adequate foreign reserves to the procurement of chemotherapeutic agents.	
Maternal, sexual and reproductive health issues and child health	Restore all vaccination services urgently, including community outreach vaccination programmes, with appropriate IPC measures.	High
	Restore antenatal and postnatal clinics urgently, with appropriate IPC measures.	
	Restore elective Caesarean sections urgently, with appropriate IPC guidance, and minimum numbers of appropriate personnel in operating theatre.	
	Restore maternal shelters, with appropriate IPC guidance, where logistical barriers to accessing facilities occur.	
	Restore family planning clinics and other services relevant to Maternal, Sexual and Reproductive Health, with appropriate IPC measures. Ensure the availability of contraceptives extends to quarantine centres.	
	Promote the use of long-acting reversible contraceptives where there are no contraindications, and offer 3-6 months' supply of oral contraceptives whenever possible. Increase collaboration with existing partners to ensure continued availability of contraceptives.	
Chronic conditions	Restore outpatient consultations for patients with chronic illnesses, and wherever possible and appropriate, make use of digital health platforms.	High
	Offer prescription refills for 3-6 months whenever appropriate.	
	Make use of existing community structures such as village healthworkers and Community ART Refill Groups to ensure continuity of care and avoid disruptions in uptake of medicines.	

## Conclusion

Strategies to deal with the pandemic effectively whilst protecting the population from other problems require a holistic approach and wider consultative forums with various stakeholders involved in healthcare delivery. Thus, authorities must include public health experts from various backgrounds to optimise interventions whilst protecting the population from other public health challenges, and especially to protect patients from avoidable delays in accessing essential health services.
